# Photosensitivity Episodes Related to Skin Color in People Treated With Dual‐Wavelength Low Power Laser Therapy: A Retrospective Cohort Study

**DOI:** 10.1111/phpp.70042

**Published:** 2025-08-10

**Authors:** Caio Bruno Tolentino de Brito, Caio Camargo Calarga, Fabianne Soares Lima, Patrícia Moreira Freitas, Alyne Simões

**Affiliations:** ^1^ School of Dentistry University of São Paulo São Paulo Brazil

**Keywords:** cohort study, low‐level light therapy, photobiomodulation, photomedicine, photosensitivity disorders, phototherapy, skin

## Abstract

**Background/Purpose:**

The therapeutic use of low‐level lasers aims to promote tissue photobiomodulation without raising the local temperature. However, in darker‐skin patients, melanin can increase temperature and cause adverse effects such as photosensitivity, especially in protocols with higher irradiances, such as those used in dual irradiations. Therefore, through a retrospective cohort, this project assesses the relationship between self‐reported skin color and the frequency of photosensitivity in patients treated with simultaneous dual irradiation protocols at the School of Dentistry of the University of São Paulo.

**Methods:**

Data from patients treated with lasers between 2013 and 2023 were retrieved. Patients were divided into two groups based on skin color and assessed for photosensitivity after simultaneous irradiation with red and infrared wavelengths. Statistical analyses investigated the relationship between photosensitivity, skin color, patient characteristics, and delivered energy.

**Results:**

Self‐reported skin color was associated with photosensitivity, with dark‐skinned patients having a three times higher risk of heating or thermal injury. The risk of clinically visible thermal injuries was nearly 12 times higher for this group compared to those with lighter skin. There was no correlation between the energy used and the degree of photosensitivity, nor was there a significant difference in energy between patients with and without photosensitivity.

**Conclusion:**

Self‐reported skin color was associated with photosensitivity occurrence during simultaneous laser wavelengths irradiation. Further studies are needed to investigate the mechanisms underlying the relation between melanin and irradiance. Finally, individualized photobiomodulation protocols are needed to ensure patient safety and optimize the therapy's expected effects.

## 1 Introduction

The therapeutic use of lasers is grounded in the interaction of electromagnetic waves with biological tissues, leading to two primary effects: photobiomodulation (commonly associated with low‐power devices) and photothermal effects (typically produced by high‐power equipment). These effects have led to the widespread adoption of lasers in various healthcare fields, including dermatology, ophthalmology, physiotherapy, dentistry, and general surgery [1, 2, 3].

Numerous studies have aimed to understand how biological tissues utilize light energy to achieve benefits like accelerated tissue repair, inflammation modulation, and pain relief. However, the effectiveness of these therapies in clinical practice can be influenced by a variety of factors, such as age, skin color, body mass, and overall health status of the patient, as well as the technique and experience of the professional, equipment specifications, and the dosimetric parameters used [4]. In addition to potentially affecting the expected outcomes of photobiomodulation, these factors can sometimes result in adverse effects. These may include noticeable increases in local temperature, which can lead to episodes of photosensitivity, generating discomfort, and in some cases, burn injuries [3, 4, 5, 6, 7].

Regarding skin color, melanin—the primary pigment responsible for determining it—plays a crucial role in how laser light interacts with the skin [8]. Melanin can impede the transmission of photons to deeper tissue targets, acting as both a competing chromophore and a scattering agent for the emitted light [6]. As a chromophore, melanin absorbs light across a wide spectrum, from the near infrared (NIR) to the ultraviolet range, with peak absorption occurring at wavelengths below 500 nm.

Although melanin's absorption decreases to its minimum in the NIR range (above 800 nm), it remains significant in the red‐light spectrum (600–700 nm), which is commonly used in many low‐power lasers [6, 9]. As a result, under some specific conditions, melanin can absorb a substantial portion of the radiated energy in these wavelengths, being responsible for the rise in local temperature. This phenomenon has been confirmed by previous studies that examined the association between skin color and the heat generated by laser treatments [5, 6].

In the context of dosimetric parameters, an innovative approach in PBMT involves using infrared and red wavelengths simultaneously. This technique seeks to combine the therapeutic benefits of both wavelengths [10, 11]. However, existing literature underscores the importance of careful consideration of these protocols due to potential adverse effects. One possible explanation is that dual irradiation can increase the power density within the treated area [11, 12]. This elevation in irradiance may heighten sensitivity in some individuals, particularly depending on the administered dosage and target tissue.

Given these considerations, when employing dual irradiation protocols on the skin, in addition to the possibility of the laser device amplifying irradiance, melanin's role as a chromophore would also be an aggravating factor for the increase in energy concentration at the irradiation site. Our hypothesis is that this would potentially result in increased temperatures and episodes of photosensitivity, mainly in people with darker skin. Therefore, the objective of this study was to assess—through a retrospective cohort—the association between self‐reported skin color and the incidence of photosensitivity cases in patients treated with dual irradiation protocols at the Special Laser Laboratory in Dentistry (LELO FOUSP).

## 2 Materials and Methods

This research was approved by the research ethics committee of the School of Dentistry of the University of São Paulo (#6.160.680). Data used in this study were extracted from the records of patients treated between 2013 and 2023 at the LELO FOUSP. Records were specifically selected for patients who underwent treatment involving simultaneous irradiation with red (660 nm) and infrared (808 nm) wavelengths emitted from the same device (side‐by‐side optical configuration), each with an emission power of 100 mW. Information retrieved included sex, age, diagnosis (reason for treatment), self‐reported skin color, medications in use, maximum irradiated energy used (in joules), and reports of photosensitivity, categorized into grade 0 (nothing), grade I (heating sensation) and grade II (burns).

Patients were divided into two groups based on their self‐reported skin color: Group 1 comprised participants with lighter skin tones (white and Asians), and Group 2 included participants with darker skin tones (indigenous, mixed‐race, and black). Patients' records that lacked information about self‐reported skin color or included fewer than two laser therapy sessions were excluded from the study.

Statistical analyses were performed using Jamovi (version 2.4.8.0) [13, 14]. The chi‐square test was employed to assess the association between photosensitivity occurrence and variables, such as medication use, age, and group classification. Binomial logistic regression was utilized to examine the correlation between group classification and photosensitivity. The Mann–Whitney test was applied to compare the median maximum energy used across groups and between patients who did or did not experience photosensitivity. Finally, the Pearson correlation test was conducted to evaluate the relationship between maximum energy used and the severity of photosensitivity.

## 3 Results

**FIGURE 1:**
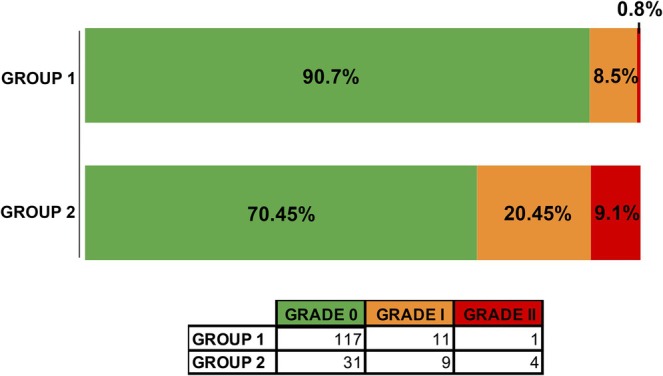
Percentage and number of photosensitivity cases in the groups according to each grade (grade 0—nothing; grade I—heating sensation; grade II—burns).

A total of 173 patients participated in this research, including 114 women and 49 men. Group 1 consisted of 129 participants, while Group 2 had 44 participants. The ages of the participants ranged from 8 to 76 years, with a mean age of 40.3 years (SD ±14.3). Among the types of conditions treated were included patients with facial paralysis (21) and paresthesia (152). Among all participants, 25 suffered episodes of photosensitivity: 20 of them were classified as grade I and 5 as grade II (Figure 1). The laser parameters used in the treatments are shown in Table 1.

**TABLE 1:** Laser parameters.

Parameter	Description
Manufacturer	DMC (São Carlos, Brazil)
Wavelength	660 nm and 808 nm
Output power	100 mW for each laser beam
Beam profile	Gaussian
Operation mode	Continuous
Spot size	0.028 cm^2^
Irradiance	3.57 W/cm^2^
Energy density	Between 35.7 J/cm^2^ and 214.2 J/cm^2^, considering protocol customization
Exposure time	Between 1 and 3 s per point, considering protocol customization

The chi‐square test demonstrated a significant association between the groups and the occurrence of photosensitivity (*p* < 0.001). Group 2 had a risk of experiencing photosensitivity approximately three times higher than Group 1 (RR = 3.18, 95% CI: 1.57–6.43). Additionally, the darker skin colors were associated with a 20% increase in the occurrence of photosensitivity events among the participants (AR = 0.202, 95% CI: 0.0586–0.346). The logistic regression analysis also showed a significant correlation between the self‐reported skin color and the occurrence of photosensitivity (*p* = 0.002). Participants in Group 2 had a risk approximately four times higher than that of Group 1 for experiencing photosensitivity (Table 2).

**TABLE 2:** Binominal logistic regression between the groups and photosensitivity occurrence.

						95% Confidence interval		Model fit measures		
Predictor	Estimate	SE	*Z*	*p*	Odds ratio	Lower	Upper	Deviance	AIC	*R* ^2^_McF_
Intercept	0.869	0.330	2.63	0.009	2.38	1.25	4.56	133	137	0.0676
Group										
1–2	1.408	0.448	3.14	0.002	4.09	1.70	9.85			

*Note:* Estimates represent the log odds of “Photosensitivity = NO” vs. “Photosensitivity = YES”.

The Mann–Whitney test for non‐parametric samples did not show a significant difference in maximum energy usage between patients with or without reported photosensitivity (*p* = 0.54), although Group 2 had a higher median maximum energy compared to Group 1 (*p* = 0.001) (Table 3). Additionally, the Pearson test revealed no significant correlation between the increase in maximum energy and the severity of photosensitivity (*p* = 0.180).

**TABLE 3:** Maximum energy used.

Group	*N*	Mean	Median	SD	SE
1	129	2.96	3.00	0.934	0.0826
2	44	3.45	4.00	0.730	0.110
Photosensitized	25	3.28	3.00	0.678	0.136
Not photosensitized	148	3.05	3.00	0.942	0.0777

Analyzing only the most severe photosensitivity episodes, the Fisher's Exact test showed a significant association (*p* = 0.015) between the groups and the occurrence of grade II photosensitivity. Group 2 had approximately 12 times the risk of experiencing burns compared to Group 1 (RR 11.7, 95% CI: 1.35–5.102). Regarding the logistic regression analysis evaluating the correlation between the self‐reported skin color and the occurrence of grade II photosensitivity, the result showed that participants in Group 2 were nearly 13 times more likely to experience burns than those in Group 1 (*p* = 0.024) (Table 4).

**TABLE 4:** Binominal logistic regression between the groups and burns occurrence.

						95% Confidence interval		Model fit measures			
Predictor	Estimate	SE	*Z*	*p*	Odds ratio	Lower	Upper	Model	Deviance	AIC	*R* ^2^_McF_
Intercept	2.30	0.524	4.39	< 0.001	10.00	3.58	27.9	1	38.5	42.5	0.150
Group											
1–2	2.55	1.133	2.25	0.024	12.80	1.39	117.8				

*Note:* Estimates represent the log odds of “BURN = NO” vs. “BURN = YES”.

There was no association between photosensitivity episodes and sex, age, or non‐specific use of medication. No single medication appeared with sufficient frequency to establish associations between photosensitivity and the use of any specific drug, or to perform more complex analyses. However, it is noteworthy that two patients from Group 2 who experienced photosensitivity were using Sertraline, a medication previously identified as photosensitive in other studies [15].

## 4 Discussion

As noted in previous studies, skin color and irradiance are factors that can impact the efficacy and safety of PBMT [5, 6, 16]. Given this, our initial hypothesis was that, when employing dual irradiation protocols on the skin, besides the higher irradiance, melanin's role as a chromophore would also be an aggravating factor for the increase in absorption at the irradiation site. This would increase temperatures and episodes of photosensitivity, mainly in people with darker skin. Our results support this hypothesis, since we found a positive correlation between self‐reported skin color and the risk of photosensitivity in patients submitted to dual irradiation protocols.

The relationship between skin color and temperature increase during the use of low‐power lasers has already been evaluated in the literature, including through clinical trials [5, 6]. Joensen et al. [5], for example, conducted a study applying doses ranging from 2 to 12 J using NIR lasers with 60 and 200 mW output powers—1.67 and 6.37 W/cm^2^, respectively—to compare temperature increases across different skin colors. Their findings revealed significant differences in temperature rise for all scenarios, with darker‐skinned individuals experiencing the greatest increases. Notably, when using the higher‐powered protocol, 62% of the dark skin patients had to interrupt the study due to substantial temperature increases. Surprisingly, one of the patients scored a temperature above 53°C at the irradiation site, while irradiated with an energy of 6 J.

Similar to these findings, Souza‐Barros et al. [6] also observed temperature differences between light‐skinned and dark‐skinned patients when using the same energy doses in different irradiations and wavelengths. The authors used red (636 nm, 0.192 W/cm^2^) or infrared (808 nm, 0.214 W/cm^2^) lasers and, despite the differences in protocols, the highest temperature increase did not exceed 3°C (observed in the dark‐skinned group), and none of the patients noticed any thermal effect, similar to what was observed with patients irradiated using Joensen's lower power protocol.

In our study, patients who self‐identified as Black, mixed race, or Indigenous—groups with characteristically darker skin—were approximately three times more likely to experience heat or burn injuries during treatment, compared to individuals with lighter skin tones. These results underscore the necessity for heightened caution when employing PBMT higher‐irradiance protocols, such as dual irradiation, particularly on patients with darker skin. Depending on the energy required for treatment, it may be advisable to reduce the device's output power. Alternatively, if reducing the power is not feasible, opting for single wavelength emission protocols may be a safer approach to minimize the risk of adverse effects in these cases. Additionally, it is crucial for manufacturers of laser devices to be aware of these findings and to avoid producing equipment with excessively high output powers. The American National Standards Institute's Z136.1 Guidelines for Safe Use of Lasers, for example, recommends irradiances below 0.2 W/cm^2^ for the red spectrum and 0.4 W/cm^2^ for the NIR spectrum when the target tissue is skin, regardless of color [6, 17].

Additionally, the size of the equipment's spot can also influence photosensitivity events, as irradiance depends on the ratio between the laser output power and the applicator area. Therefore, even low power devices can produce high irradiances if their spots are small. However, simply using larger applicators may not be the best approach, as studies have shown that the output power has a Gaussian distribution within the spot, with most of the energy being delivered to the central third of the laser beam. Consequently, increasing the applicator size might result in excessive irradiance in the center while underexposing the periphery [3, 18, 19].

**FIGURE 2:**
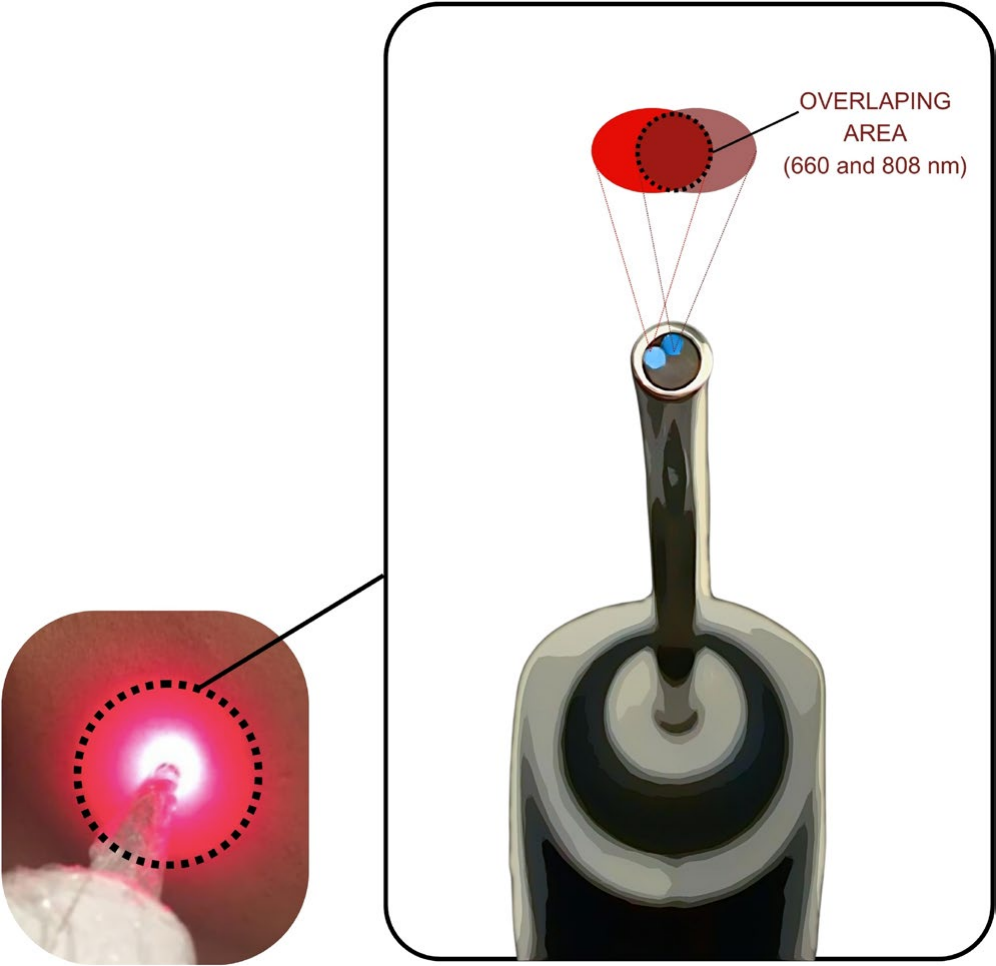
Arrangement of the beams in the tip of the laser device used for dual wavelength LLLT treatments at LELO‐USP.

In the case of the device used to treat the patients in this study, its spot had a small size (0.028 cm^2^) and featured two beams (red and infrared), each with an output power of 100 mW (Figure 2). Even though separated, the optic fibers are side by side, and tests carried out by the authors using a power meter (MMO, São Carlos, Brazil) revealed that the maximum power was 180 mW when both red and infrared wavelengths were activated. Therefore, the power density at the application site during simultaneous irradiation protocols would be much greater than that delivered by single irradiation protocols.

It is known that, upon penetrating the tissue, the irradiance tends to decrease due to reflection and scattering phenomena [18, 19]. However, similar to what was described by Cotomacio et al. [11], we assume that the intersection created by the scattering of the two side‐by‐side beams may lead to an overlapping energy area, resulting in a higher dose than that emitted by each independent beam. Our hypothesis is that this could significantly increase the local temperature, which may be even more exacerbated by a higher concentration of melanin. It is important to highlight that this hypothesis is based only on theoretical assumptions. A limitation of our study is the absence of specific methods to directly confirm these potential thermal effects. Moreover, we found no studies in the literature that have performed this type of analysis with dual emission protocols to support this hypothesis. Therefore, we recommend that future research incorporate appropriate methodologies to validate this hypothesis and further elucidate the underlying mechanisms.

According to the consulted records, the clinical aspect of the thermal lesions presented by the patients included in this study was described as hypopigmented macules. The literature contains several reports of dyschromias as a secondary effect of laser dermatological procedures [20, 21, 22, 23, 24, 25, 26, 27, 28]. However, we are unaware of any previous specific reports in the literature related to low‐power laser therapies and their association with hypopigmentation. Examples of the pathogenesis mechanisms associated with high‐power laser procedures include the direct destruction of melanosomes in epidermal cells and the thermal damage caused to melanocytes, impairing melanin production and the transfer processes of melanosomes to keratinocytes. The release of inflammatory factors that signal melanocytes to suppress melanogenesis has also been associated with these lesions [22, 29, 30, 31].

Besides the fact that hypopigmentation occurs more frequently in darker skin tones, its occasional manifestation has also been previously associated with hereditary factors [29, 30, 31, 32]. This theory would possibly explain the fact that some of the patients who experienced sensitivity to the temperature increase may have also sustained thermal damage at the level of a burn; however, the injury might not have been clinically visible in the subsequent days due to a lack of genetic susceptibility.

This study has some limitations that should be considered when interpreting the results. It is notable that social factors can influence individuals to self‐classify as lighter rather than darker [33], leading to underestimation of the association between skin pigmentation and adverse reactions. By adopting a dichotomous classification of skin color into two broad categories (lighter vs. darker skin tones), specially grouping self‐reported mixed‐race and black individuals together, we aimed to minimize the misclassification bias and maintain sufficient statistical power for the analyses. Nonetheless, we acknowledge that this simplification limits the specificity of the findings.

Due to the retrospective nature of this study, self‐reported skin color was the only available variable to estimate the potential influence of pigmentation on adverse reactions. Despite its inherent subjectivity, it reflects the clinical perception of an individual's skin pigmentation. Although this perception does not capture all the physicochemical nuances involved in the interaction between skin and light radiation, it remains relevant to the expected biological effects. Future studies including objective phototype assessments are recommended to validate and expand upon these findings, avoiding the risk of misclassification bias. However, grouping patients into only two categories aimed to minimize the effects of this factor. Moreover, it should also be remembered that even an objective classification could impose certain limitations, as skin responses to radiation can vary widely. This variability can be observed even with the most commonly used method of phototype assessment, the Fitzpatrick scale, for example [34].

Despite these limitations, our study demonstrated a substantial correlation between episodes of photosensitivity and skin color in patients irradiated with simultaneous wavelength protocols. Given the importance of this correlation, further studies are needed to investigate the mechanisms behind these interactions. Furthermore, more comprehensive data on the impact of individual systemic medications and their interaction with skin pigmentation in photosensitivity risk are needed, along with more robust statistical analyses. This would help enhance patient safety and optimize the effects of photobiomodulation, aiming for more individualized and effective protocols tailored to each patient and clinical situation.

## 5 Conclusion

In conclusion, individuals with self‐reported dark skin exhibited a higher risk of photosensitivity episodes when treated with simultaneous wavelength irradiation protocols, especially considering only the most severe degree of photosensitivity (burns). Moreover, more specific research involving low power lasers is needed to further investigate the mechanisms underlying the interaction between melanin and irradiance. Finally, this work highlights the importance of individualized photobiomodulation protocols to ensure patient safety and optimize the therapy's expected effects.

## Disclosure

The authors have nothing to report.

## Conflicts of Interest

The authors declare no conflicts of interest.

## Data Availability

The data that support the findings of this study are available from the corresponding author upon reasonable request.
